# Rhamnolipid Enhances the Nitrogen Fixation Activity of *Azotobacter chroococcum* by Influencing Lysine Succinylation

**DOI:** 10.3389/fmicb.2021.697963

**Published:** 2021-07-30

**Authors:** Jin Li, Hu Pan, Hui Yang, Chong Wang, Huhu Liu, Hui Zhou, Peiwang Li, Changzhu Li, Xiangyang Lu, Yun Tian

**Affiliations:** ^1^College of Bioscience and Biotechnology, Hunan Agricultural University, Changsha, China; ^2^Institute of Agricultural Product Quality Standard and Testing Research, Tibet Academy of Agricultural and Animal Husbandry Sciences, Lhasa, China; ^3^College of Food Science and Technology, Hunan Agricultural University, Changsha, China; ^4^State Key Laboratory of Utilization of Woody Oil Resource, Hunan Academy of Forestry, Changsha, China

**Keywords:** nitrogen fixation, *Azotobacter chroococcum*, posttranslational modifications, lysine succinylation (K^Suc^), rhamnolipid

## Abstract

The enhancement of nitrogen fixation activity of diazotrophs is essential for safe crop production. Lysine succinylation (K^Suc^) is widely present in eukaryotes and prokaryotes and regulates various biological process. However, knowledge of the extent of K^Suc^ in nitrogen fixation of *Azotobacter chroococcum* is scarce. In this study, we found that 250 mg/l of rhamnolipid (RL) significantly increased the nitrogen fixation activity of *A. chroococcum* by 39%, as compared with the control. Real-time quantitative reverse transcription PCR (qRT-PCR) confirmed that RL could remarkably increase the transcript levels of *nifA* and *nifHDK* genes. In addition, a global K^Suc^ of *A. chroococcum* was profiled using a 4D label-free quantitative proteomic approach. In total, 5,008 K^Suc^ sites were identified on 1,376 succinylated proteins. Bioinformatics analysis showed that the addition of RL influence on the K^Suc^ level, and the succinylated proteins were involved in various metabolic processes, particularly enriched in oxidative phosphorylation, tricarboxylic acid cycle (TCA) cycle, and nitrogen metabolism. Meanwhile, multiple succinylation sites on MoFe protein (NifDK) may influence nitrogenase activity. These results would provide an experimental basis for the regulation of biological nitrogen fixation with K^Suc^ and shed new light on the mechanistic study of nitrogen fixation.

## Introduction

Nitrogen has been considered as a critical factor to restrict agricultural production. Biological nitrogen fixation (BNF) is a process in which diazotrophs catalyze the reduction of atmospheric N_2_ to NH_3_ through nitrogenase enzyme systems (Bloch et al., 2020), which is the main source of nitrogen uptake for crops. *Azotobacters* are the previously discovered and studied free-living, obligately aerobic diazotrophs, which have been used as nitrogen fixation fertilizer for more than a century (Das, 2019). *Azotobacter chroococcum* was isolated from soils and the rhizosphere of various plants (Lenart, 2012; Rizvi and Khan, 2018). The species of *A. chroococcum* promote crop growth and fruit development through secretion of growth hormones or nitrogen fixation (Kumar et al., 2019; Song et al., 2020). Because of the relatively low nitrogen fixation efficiency of *Azotobacters* compared with Rhizobia, which hardly satisfies the nitrogen demand of crops, the improvement of its efficiency is crucial. The use of molecular biotechnology to construct efficient nitrogen fixation strains is an effective way to improve the efficiency of nitrogen fixation (Peralta et al., 2004; Orikasa et al., 2010; Deng et al., 2018; Wang T. et al., 2018). Besides, the use of chemical materials or reagents can also improve the nitrogen fixation efficiency of *A. chroococcum*. For instance, the addition of 5% perfluorodecalin and 4 μg/ml of carbon dots can effectively improve the nitrogen fixation activity of *A. chroococcum* (Bakulin et al., 2007; Wang H. et al., 2018). The non-ionic surfactant Alk 3 could enhance the nitrogen fixation activity of *Rhizobium trtfolii* in soils (Fisher et al., 1978), but there have been no studies of surfactant on the nitrogen fixation activity of *A. chroococcum*.

Posttranslational modifications (PTMs) are the process of reversible covalent modification on the main and side chains of amino acid residues of proteins (Wagner and Payne, 2013; Gil et al., 2017). PTMs are a key feature of bacteria that have the ability to regulate the functions of proteins. PTMs mainly include acetylation, methylation, phosphorylation, ubiquitination, succinylation, crotonylation, 2-hydroxyisobutyrylation, and benzoylation. Lysine is one of the most common targets for covalent modification among the 20 amino acids of proteins (Macek et al., 2019). Lysine succinylation (K^Suc^) is a novel acyl modification and has been found widely in eukaryotes and prokaryotes (Zhang et al., 2011; Xie et al., 2012; Weinert et al., 2013), which is involved in many important metabolic processes, such as the ribosomal complex, glycolysis, gluconeogenesis, tricarboxylic acid cycle (TCA) cycle, and carbon metabolism (Xu et al., 2017; Ren et al., 2018; Zhang et al., 2019). Furthermore, BNF is a relatively complex process, and apart from the direct involvement of nitrogenase enzyme systems (Mus et al., 2018), it also requires the synergistic action of several biological processes, such as respiration, hydrogenases, electron transport, carbon metabolism, transport systems, and secretion systems. Therefore, we hypothesized that K^Suc^ may be present in diazotrophs.

In this study, we evaluated the effects of anionic surfactant sodium dodecyl sulfate (SDS), non-ionic surfactant Triton X-100 (Tr), and non-ionic biosurfactant rhamnolipid (RL) on *A. chroococcum* and found that RL could effectively improve the nitrogen fixation activity. Meanwhile, a 4D label-free quantitative proteomic analysis was conducted to explore the mechanism of RL enhancing nitrogen fixation activity. This study provides not only a new technical approach to improve the nitrogen fixation activity of diazotrophs but also a reference for K^Suc^ involved in the nitrogen fixation process.

## Materials and Methods

### Strains, Media, and Cultivation Conditions

*Azotobacter chroococcum* strain HR1 was isolated by our laboratory. The strain HR1 was cultured in nitrogen-free medium (20 g sucrose, 0.2 g MgSO_4_⋅7H_2_O, 0.1 g K_2_HPO_4_, 0.4 g KH_2_PO_4_, 0.1 g NaCl, 0.01 g FeCl_3_, 0.002 g Na_2_MoO_4_⋅H_2_O, and 1,000 ml ddH_2_O) (Hong et al., 2009) at 30°C with shaking at 100 rpm for 36 h.

### Cell Growth and Viability

The different types of surfactants (SDS, Tr, and RL) were accurately weighed and prepared in dH_2_O to form mother liquors of 10 g/l SDS, 10% Tr (*v*/*v*), and 10 g/l RL, respectively. Prior to the experiments, SDS and Tr were sterilized by heat steam and RL was filtered by 0.22 μm pore size filters. The different surfactants were added to the nitrogen-free medium, and the low and high concentrations of SDS (10 and 100 mg/l), Tr (0.025 and 0.1%), and RL (25 and 250 mg/l) could be adjusted according to experimental requirements.

The effect of different surfactants (SDS, Tr, and RL) on the growth of *A. chroococcum* was investigated using a Bioscreen C Microbiological Growth Analyser (Labsystems, Helsinki, Finland) in 100-well honeycomb plates. *A. chroococcum* cells were grown to log phase in nitrogen-free medium, and the cells were collected by centrifugation (4°C, 3,500 rpm, and 5 min), washed twice with ddH_2_O, and adjusted to a concentration of ∼10^9^ cells/ml. Each well of the honeycomb plate contained 25 μl cell suspension and 175 μl of the nitrogen-free medium with or without different surfactants (SDS, Tr, and RL). The plates were incubated at 30°C for 72 h with shaking at medium amplitude, and the OD_600_ values were recorded every 4 h. All of the experiments were conducted triplicate.

The antibacterial activity of *A. chroococcum* was evaluated by colony-forming unit (CFU) counting. The cells were treated with different surfactants (SDS, Tr, and RL). *A. chroococcum* cells were grown to log phase in nitrogen-free medium or nitrogen-free medium supplemented with different concentrations of surfactants (SDS, Tr, and RL). After that, the cells were collected and adjusted to a concentration of ∼10^9^ cells/ml. The cell suspensions were applied with a 10^8^-fold serial dilution of ddH_2_O. Onto the nitrogen-free medium plates, 200 μl cell suspensions were spread and incubated at 30°C for 36 h. The number of colonies per treatment was counted. All the experiments were tested in triplicate.

### Nitrogen Fixation Activity by Acetylene Reduction Assay

The nitrogen fixation activity was determined by acetylene reduction assay (Hardy et al., 1973). The cell suspensions were diluted with nitrogen-free liquid medium to a final OD_600_ of ∼0.3. A 5-ml aspirate of the nitrogen-free medium with or without surfactants was placed inside a serum bottle with a rubber cap, and 10% of the air in the tubes was removed and replaced with acetylene. After incubation at 30°C for 20 h with shaking (150 r/min), 100 μl of gas in these bottles was extracted, and the amount of ethylene and acetylene was determined by gas chromatography. The nitrogen fixation activity is expressed as nanomole C_2_H_4_/h/OD_600_. Data presented were representative of the means from three replicates.

The nitrogen fixation activity analysis was carried out by a SCION 456-GC (Techcomp, Beijing, China) gas chromatograph with an FID detector. An HP-PLOT capillary column (30 m × 0.32 mm × 20.00 μm) was used for the chromatographic separation, and the carrier gas was ultra high purity nitrogen. Gas flow rate and split ratios were 1.5 ml/min and 10:1, respectively. The temperatures of the inlet, oven, and detector were 200, 60, and 250°C, respectively.

### Real-Time PCR Analysis

To compare the transcript levels of *nif* genes, *A. chroococcum* cells were grown in nitrogen-free medium (with or without different surfactants) for 22 h and harvested for total RNA isolation and RT-PCR analysis. Total RNA was extracted using the TRIzol reagent, and cDNAs were synthesized using Quantscript RT Kit (Tiangen, Beijing, China). Real-time quantitative reverse transcription PCR (qRT-PCR) was performed with three independent RNA preparations using Applied Biosystems 7300 Real-Time System and detected using GoTaq^®^ qPCR Master Mix (Promega, Madison, CT, United States). The 16S RNA gene was used as a stable reference gene for the normalization of samples. The relative transcript levels were calculated by the 2^–△^^△^^CT^ method and were normalized. PCR primers for *nif* genes and 16S rRNA are shown in Supplementary Table 1.

### Protein Extraction

The *A. chroococcum* cells treated with RL (250 mg/l) and without RL were grinded by liquid nitrogen and resolved in lysis buffer [8 M urea, 50 mM nicotinamide (NAM), 3 μM trichostatin A (TSA), and 1% Protease Inhibitor Cocktail]. Cells were sonicated three times on ice using a high-intensity ultrasonic processor (Scientz, Ningbo, China). The supernatant was collected by centrifugation at 12,000 × *g* for 10 min at 4°C. Protein concentration was determined using a BCA kit (Tiangen, Beijing, China).

### Western Blotting Analysis

The protein extracts (20 μg) were separated by 12% SDS-PAGE gel and then transferred to PVDF membranes. The membrane was incubated with pan anti-succinyl lysine antibody (1:1,000, PTM Biolabs, Hangzhou, Zhejiang, China) at 4°C overnight. The membrane was washed five times with Tris-buffered saline with Tween 20 (TBST) (25 mM Tris–HCl, pH 8.0; 125 mM NaCl; and 0.1% Tween 20) containing 5% (*w*/*v*) BSA for 10 min and subsequently incubated with goat anti-mouse IgG (1:1,000, Thermo Fisher Scientific, Waltham, MA, United States) at an ambient temperature for 2 h. After the membrane was washed with TBST buffer, the SuperSignal West Pico kit (Pierce, Dallas, TX, United States) was used for visualizing.

### Trypsin Digestion and HPLC Fractionation

The collected proteins were added with dithiothreitol (DTT, 5 mM), reduced at 56°C for 30 min, and then alkylated with iodoacetamide (11 mM) for 15 min at an ambient temperature in the dark. The proteins were then diluted by adding 100 mM tetraethylammonium bromide (TEAB) to a urea concentration less than 2 M and digested with trypsin (Promega, Madison, WI, United States) to a trypsin/protein ratio of 1:50 (*w*/*w*) at 37°C overnight. After digestion, peptides were fractionated into fractions using a Thermo BetaSil C18 column (250 mm × 10 mm × 5 μm) with a gradient of 8% to 32% acetonitrile (pH 9.0) over 60 min into 60 fractions. Then, the peptides were combined into six fractions and dried by vacuum centrifugation.

### Affinity Enrichment and Liquid Chromatography With Tandem Mass Spectrometry Analysis

The peptides were dissolved in NETN buffer (100 mM NaCl, 1 mM EDTA, 50 mM Tris–HCl, and 0.5% NP-40, pH 8.0) and mixed with conjugated pan anti-succinyl lysine agarose antibody beads (PTM Biolabs, Hangzhou, Zhejiang, China) at 4°C overnight with gentle shaking. The beads were washed four times with NETN buffer and twice with dH_2_O subsequently. The peptides bound were eluted from the beads with 0.1% trifluoroacetic acid (TFA) and desalted with C18 Zip-Tips (Millipore, Billerica, MA, United States).

Liquid chromatography with tandem mass spectrometry (LC-MS/MS) analysis was performed at PTM Biolabs, Hangzhou, Zhejiang, China. Briefly, the enriched peptides were dissolved in buffer A [0.1% (*v*/*v*) formic acid in dH_2_O] and loaded directly onto a home-made reversed-phase analytical column (15-cm length and 75-mm diameter) using an EASY-nLC 1000 UPLC system (Thermo Fisher Scientific, Waltham, MA, United States) for elution. The gradient was 6–22% buffer B (0.1% formic acid in 98% acetonitrile) for 44 min, 22–30% for 12 min, 30–80% for 2 min, and finally held at 80% for 2 min. The flow rate of the column was maintained at 300 nl/min.

The resultant peptides were then analyzed by MS/MS in timsTOF Pro (Bruker, Billerica, MA, United States) coupled online to the UHPLC. The electrospray voltage was set at 1.4 kV. Precursors and fragments were analyzed at the TOF detector, with an MS/MS scan range set as 100–1,700 *m*/*z*. The Bruker timsTOF Pro was operated in parallel accumulation serial fragmentation (PASEF) mode. Precursors with charge states 0–5 were selected for fragmentation, and 10 PASEF-MS/MS scans were acquired per cycle. The dynamic exclusion was set to 24 s. Three biological replicates were performed.

### Proteomics Database Search

The resulting MS/MS data were processed using the MaxQuant search engine (v.1.6.6.0). Tandem mass spectra were searched against the UniProt *A. chroococcum* (strain NCIMB 8003) database downloaded from NCBI (4,584 protein sequences, released 2015) and concatenated with a reverse decoy database. Trypsin/P was specified as a cleavage enzyme and up to four missing cleavages. The mass tolerance of precursor ions for the first search and main search was set as 40 ppm, and the mass tolerance for fragment ions was 0.04 Da. Carbamidomethyl on Cys was specified as a fixed modification, and acetylation on protein N-terminal, succinylation, deamidation, and oxidation on methionine were specified as variable modifications. Peptide and protein false discovery rate (FDR) was adjusted to <1%, and the minimum score of modified peptides was more than 40 (Cox and Mann, 2008).

### Bioinformatics Analysis

K^Suc^ sites (10 amino acids upstream and downstream of the motif site) were analyzed using MoMo (Chou and Schwartz, 2011). Gene Ontology (GO) annotations of the succinylated proteins were compared against the UniProt-GOA database^1^ using InterProScan (v.5.14–53.0). The subcellular localization prediction analysis of succinylated proteins was using CELLO. The GO, domains, and Kyoto Encyclopedia of Genes and Genomes (KEGG) were used for functional enrichment analyses. Two-tailed Fisher’s exact tests were applied to each category to obtain *p* values for the analysis of functional enrichment, the terms (*p* < 0.05) were considered significant. According to the succinylation quantification analysis, a 1.5-fold change was set as the differential expression multiple.

The further hierarchical clustering was based on differentially succinylated protein functional classification (GO, domains, and KEGG). Those categories, which were at least enriched in one of the clusters with *p* value <0.05, were filtered. This filtered *p* value matrix was transformed by the function *x* = −log10 (*p* value). These *x* values were *z*-transformed for each functional category. These *z* scores were then clustered by one-way hierarchical clustering (Euclidean distance and average linkage clustering) in Genesis. Protein–protein interaction (PPI) networks were performed by STRING database (Szklarczyk et al., 2011) and visualized by Cytoscape v. 3.7.2 (Shannon et al., 2003). The interactions that had confidence scores of 0.7 (high confidence) were fetched.

## Results

### The Effects of Surfactants on *A. chroococcum*

**FIGURE 1:**
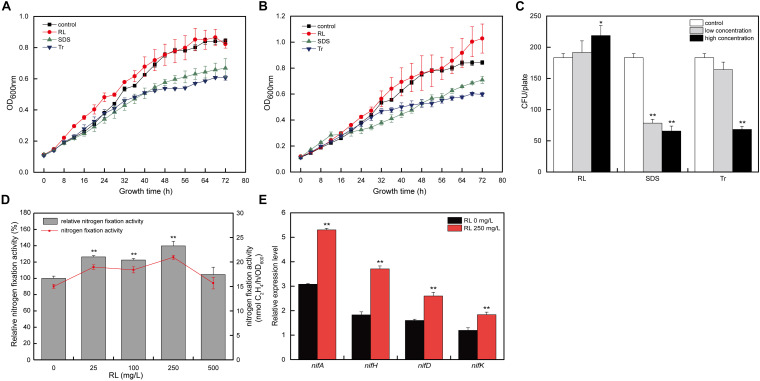
The effects of different surfactants on *A. chroococcum*. The growth curves of *A. chroococcum* cells were treated with **(A)** low concentrations of surfactant (10 mg/l SDS, 0.025% Tr, and 25 mg/l RL) and **(B)** high concentrations of surfactant (100 mg/l SDS, 0.1% Tr, and 250 mg/l RL), respectively. **(C)** The CFU analyses of *A. chroococcum*. The cells were incubated in nitrogen-free medium (control) or nitrogen-free medium added with both low and high concentration of SDS (10 and 100 mg/l), Tr (0.025 and 0.1%), and RL (25 and 250 mg/l), respectively. **(D)** The nitrogen fixation activity of *A. chroococcum* with different concentrations of RL (0, 25, 100, 250, and 500 mg/l). Relative nitrogen fixation activity of each sample represents 100% that of *A. chroococcum* cells treated without RL. **(E)** Relative transcript levels of *nif* genes in *A. chroococcum* with or without RL (250 mg/l). (*p < 0.05; **p < 0.01). SDS, sodium dodecyl sulfate; Tr, Triton X-100; RL, rhamnolipid; CFU, colony-forming unit.

To study the effects of surfactants (RL, SDS, and Tr) on *A. chroococcum*, the growth curves and survival rate of *A. chroococcum* under low and high concentrations of surfactants were determined. As shown in Figures 1A,B, the growth of *A. chroococcum* was significantly inhibited by both low and high concentrations of SDS and Tr. The effects of low concentrations of RL were comparable to the control. However, high concentrations of RL promoted the growth of *A. chroococcum* with the extension of the incubation time. At the same time, the CFU were used to validate the survival rate of *A. chroococcum* cells with different surfactants, and the result indicated that SDS and Tr had obvious antibacterial effects on *A. chroococcum* cells (Figure 1C).

Because RL showed a positive effect on the growth and activity of *A. chroococcum*, and the survival of *A. chroococcum* is an important prerequisite for nitrogen fixation, RL should be applied in nitrogen fixation. To further investigate the effect of RL on *A. chroococcum*, different concentrations of RL (0, 25, 100, 250, and 500 mg/l) were selected to determine its effects on nitrogen fixation activity. As compared with the control, different concentrations of RL could improve the nitrogen fixation activity of *A. chroococcum*, and the effect of 250 mg/l of RL (increased by 39.7%) was the most significant (Figure 1D).

The expression and synthesis of nitrogenase rely on the coordinated regulation of a series of genes (Curatti et al., 2005), which mainly include the positive regulation gene (*nifA*) and the structure gene (*nifHDK*) (Ribbe et al., 2014). To investigate the mechanisms of the enhancement of nitrogen fixation activity of *A. chroococcum* by RL, the effects of RL on the transcript levels of nitrogen-fixing enzyme genes were studied. The transcript levels of nitrogen fixation genes (*nifA*, *nifH*, *nifD*, and *nifK*) of *A. chroococcum* upon 250 mg/l of RL were significantly higher than those of the control (Figure 1E). The results indicated that RL could increase the transcript levels of these nitrogen fixation genes.

### Identification of K^Suc^ in *A. chroococcum*

**FIGURE 2:**
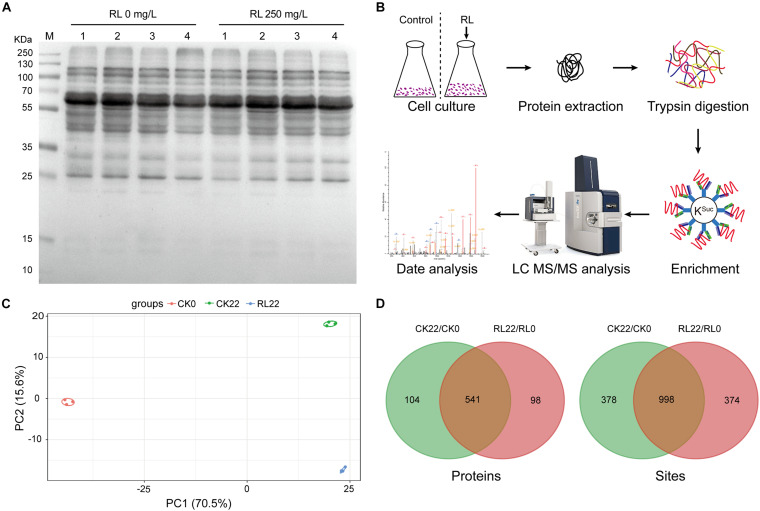
Identification of succinylation in *A. chroococcum*. **(A)** Detection of succinylated proteins using the pan anti-succinyl lysine antibody in *A. chroococcum* treated with or without RL (250 mg/l) for 0 h (lane 1), 4 h (lane 2), 12 h (lane 3), and 22 h (lane 4), respectively. **(B)** The workflow of an integrated strategy for global mapping of K^Suc^ in *A. chroococcum*. **(C)** PCA of K^Suc^. **(D)** Survey of succinylated proteins and K^Suc^ sites. CK0, *A. chroococcum* cells were treated without RL for 0 h; CK22, *A. chroococcum* cells were treated without RL for 22 h; RL0, *A. chroococcum* cells were treated without RL for 0 h; RL22, *A. chroococcum* cells were treated with RL for 22 h. K^Suc^, lysine succinylation; PCA, principal component analysis.

BNF was involved in multiple metabolic pathways (e.g., respiration, electron transport, carbon metabolism, nitrogen metabolism, etc.), and succinylated proteins play regulatory roles in various metabolic processes as previously described. Thus, the *A. chroococcum* cells were treated with RL (250 mg/l) and without RL, and the protein extracts were investigated using anti-succinyl lysine antibody by the western blotting assay. The results showed stronger reactions to the anti-succinyl lysine in all treatments, indicating the presence of K^Suc^ in *A. chroococcum* (Figure 2A). To identify the K^Suc^ sites in *A. chroococcum*, we chose 0 h (RL0) and 22 h (RL22) of 250 mg/l of RL treatments and without RL treatment as the control. Figure 2B shows the workflow of a mass spectrometry-based high-throughput proteomics approach to the identification of K^Suc^. In order to ensure the reliability of the data, the samples were tested for reproducibility using principal component analysis (PCA). The degree of aggregation between each set of replicates was good, which indicated a good reproducibility between each set of replicates samples (Figure 2C). A total of 5,008 K^Suc^ sites on 1,376 proteins were yielded (Supplementary Table 2). There were 1,376 K^Suc^ sites on 645 proteins and 1,372 K^Suc^ sites on 639 proteins found in CK22/CK0 and RL22/RL0, respectively (Figure 2D and Supplementary Table 3). The results indicated that a large number of succinylated proteins were presented in *A. chroococcum* and RL may alter the K^Suc^ level. These results imply that K^Suc^ is likely linked to nitrogen fixation in *A. chroococcum*, although the mechanism for this is unclear.

### Analyses of the Sites of K^Suc^ in *A. chroococcum*

**FIGURE 3:**
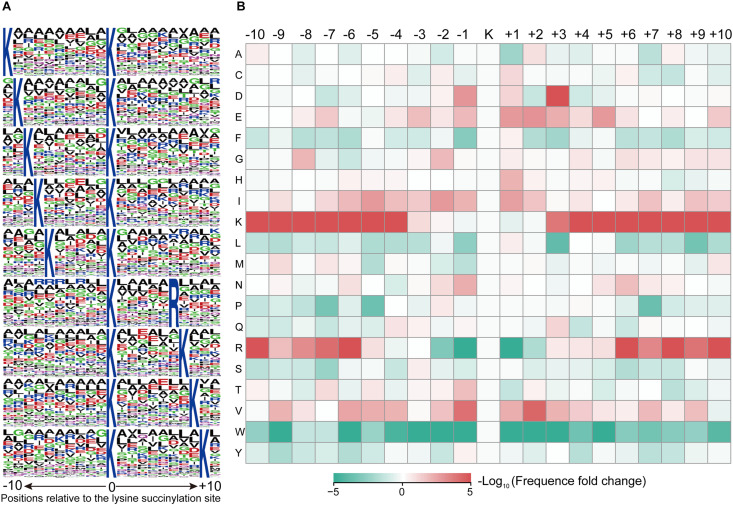
MoMo analysis of the lysine-succinylated sites. **(A)** Sequence probability of significantly enriched K^Suc^ motifs for ± 10 amino acids around the K^Suc^ sites. **(B)** Heat map of the sequence motif of K^Suc^ sites. The colors represent the enrichment (red) or depletion (green) of amino acids at specific positions.

To identify the motifs of K^Suc^, lysine (K) was frequently found both upstream and downstream of K^Suc^, while arginine (R) was frequently found downstream of K^Suc^ (Figure 3A and Supplementary Table 4). A total of nine conserved motifs were ^∗^K^∗^K^Suc*^, ^∗^K^Suc*^R^∗^, and ^∗^K^Suc*^K^∗^ [asterisk (^∗^) indicates multiple random amino acid residues]. To further assess the enrichment or depletion of specific amino acids neighboring the K^Suc^ sites, the basic amino acids [lysine (K) and arginine (R)] were significantly enriched surrounding K^Suc^ sites, but R was greatly depleted at the −1 and +1 positions, respectively. Moreover, the acidic amino acid aspartic acid (D) was enriched in positions +3 of the K^Suc^ sites, and aliphatic amino acid valine (V) was significantly enriched at the −1 and +2 positions (Figure 3B). Conserved amino acid sequences surrounding the K^Suc^ sites suggested the preference of enzymes that catalyze the succinylation modifications.

### GO and Subcellular Localization of Succinylated Proteins

**FIGURE 4:**
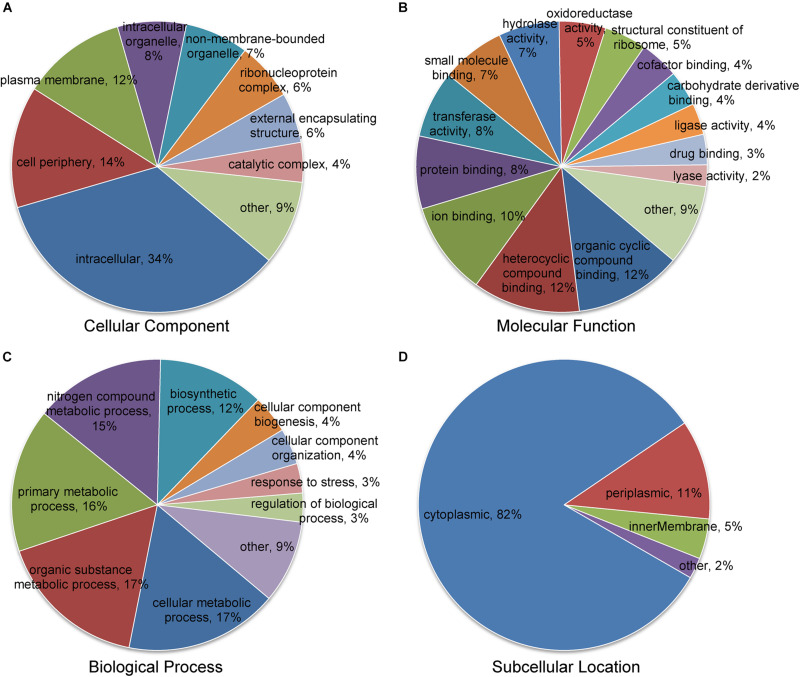
Classification of the succinylated proteins based on Gene Ontology (GO) and subcellular location. **(A)** Classification of the succinylated proteins based on the cellular component. **(B)** Classification of the succinylated proteins based on the molecular function. **(C)** Classification of succinylated proteins based on the biological process. **(D)** Subcellular location classification of the succinylated proteins.

For the purpose of understanding the biological functions of succinylated proteins in *A. chroococcum*, GO classification of all succinylated proteins was performed (Figure 4 and Supplementary Table 5). In cellular composition, intracellular (34%), cell periphery (14%), and plasma membrane (12%) were accounted for the major part of succinylated proteins (Figure 4A). In terms of molecular function, 60 and 26% of succinylated proteins were associated with binding function and catalytic activity, respectively (Figure 4B). For biological process, 65% of succinylated proteins were associated with metabolism, among which 15% of succinylated proteins were involved in the nitrogen compound metabolic process (Figure 4C). Further analysis showed that the major classes were similarly to RL treatments, but the number of succinylated proteins was different from each individual component. In addition, 21 succinylated proteins associated with intrinsic components of the membrane were present in CK22/CK0, while 35 succinylated proteins associated with response to abiotic stimulus were present in RL22/RL0. The results showed that the level of K^Suc^ in *A. chroococcum* was altered in response to RL (Supplementary Figure 1 and Supplementary Tables 6, 7).

The subcellular localization analysis indicated that the succinylated proteins in *A. chroococcum* were predominantly located in cytoplasm (82%), followed by periplasm (11%), inner membrane (5%), and other subcellular components (2%) (Figure 4D). The majority of succinylated proteins were involved in intracellular material transport, metabolism, and cellular activity, which suggested that the succinylated proteins played an important role in the growth and development of *A. chroococcum*.

### Enrichment-Based Clustering Analysis of Succinylated Proteins

**FIGURE 5:**
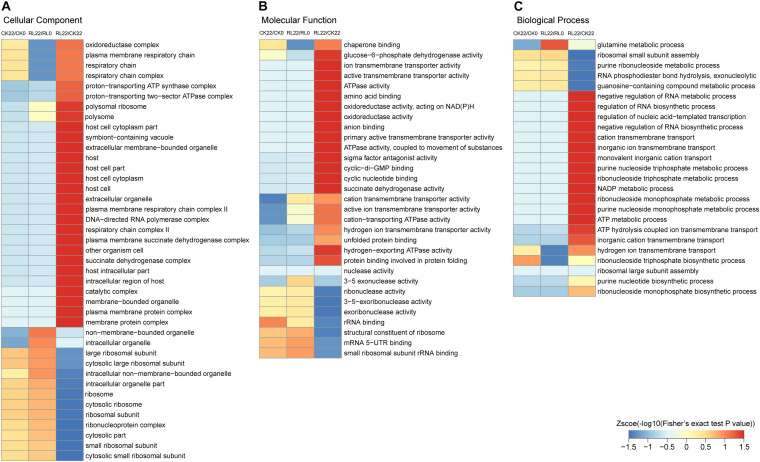
Gene Ontology enrichment-based clustering analysis of the succinylated proteins. **(A)** Cellular component analysis. **(B)** Molecular function analysis. **(C)** Biological process analysis.

To reveal the mechanisms of the enhancement of nitrogen fixation activity in *A. chroococcum* by RL, GO, protein domain, and KEGG pathway enrichment-based clustering analyses were performed (Figure 5 and Supplementary Table 8).

Gene Ontology enrichment-based clustering analysis showed various degrees of enrichment of succinylated proteins in a different category. In the cellular component category, the enrichment of succinylated proteins in CK22/CK0 and RL22/RL0 was similar, and only a few succinylated proteins were significantly different, such as oxidoreductase complexes, intracellular organelles, and those associated with the respiratory chain. However, the succinylated proteins were mainly enriched in components that were related to cellular respiration and membranes in RL22/CK22 (Figure 5A). The enrichment analysis of the molecular function category revealed significant differences in succinylated proteins, which were associated with the transmembrane activity and 3–5 exonuclease activity in CK22/CK0 and RL22/RL0; the succinylated proteins were significantly enriched in terms that were related to energy metabolisms in RL22/CK22, such as glucose-6-phosphate dehydrogenase activity, ATPase activity, oxidoreductase activity, and succinate dehydrogenase activity (Figure 5B). In the biological process category, succinylated proteins were significantly different in glutamine metabolism, hydrogen ion transport across membranes, and ribonucleoside triphosphate biosynthesis in CK22/CK0 and RL22/RL0; the enriched succinylated proteins were mainly involved in the cellular nucleic acid metabolic process in RL22/CK22, such as ion transmembrane transport and energy metabolic process (Figure 5C).

**FIGURE 6:**
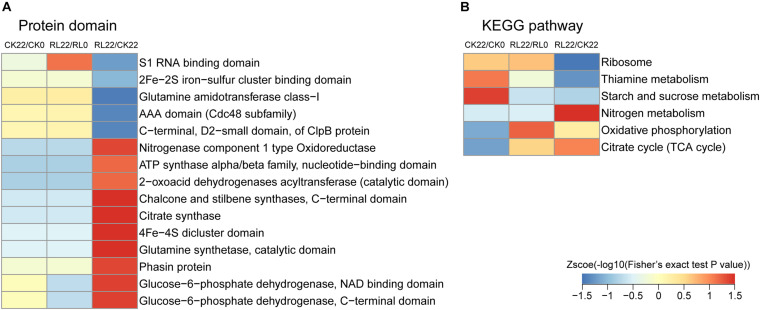
Functional enrichment-based clustering analysis of the quantified proteins. **(A)** Protein domain. **(B)** Kyoto Encyclopedia of Genes and Genomes (KEGG) pathway.

The domain enrichment analysis of succinylated proteins indicated that the majority of protein domains were similarly enriched in CK22/CK0 and RL22/RL0. However, the enriched protein domains mainly relate to nitrogenase (nitrogenase component type 1 oxidoreductase and 4Fe-4S dicluster domain) and energy metabolism (citrate synthase, glucose-6-phosphate dehydrogenase, NAD-binding domain; glucose-6-phosphate dehydrogenase, C-terminal domain; and ATP synthase alpha/beta family, nucleotide-binding domain) in RL22/CK22, followed by 2-oxoacid dehydrogenases acyltransferase, catalytic domain; chalcone and stilbene synthases, C-terminal domain; and glutamine synthetase, catalytic domain (Figure 6A). In the KEGG enrichment analysis, the succinylated proteins were mainly enriched in the ribosome, thiamine metabolism, and starch and sucrose metabolism in CK22/CK0. The ribosome, oxidative phosphorylation, and citrate cycle (TCA cycle) were the most dominant pathways in RL22/RL0. Conversely, the nitrogen metabolism, oxidative phosphorylation, and TCA cycle were significantly enriched in RL22/CK22 (Figure 6B).

### Protein Interaction Networks of Succinylated Proteins in *A. chroococcum*

**FIGURE 7:**
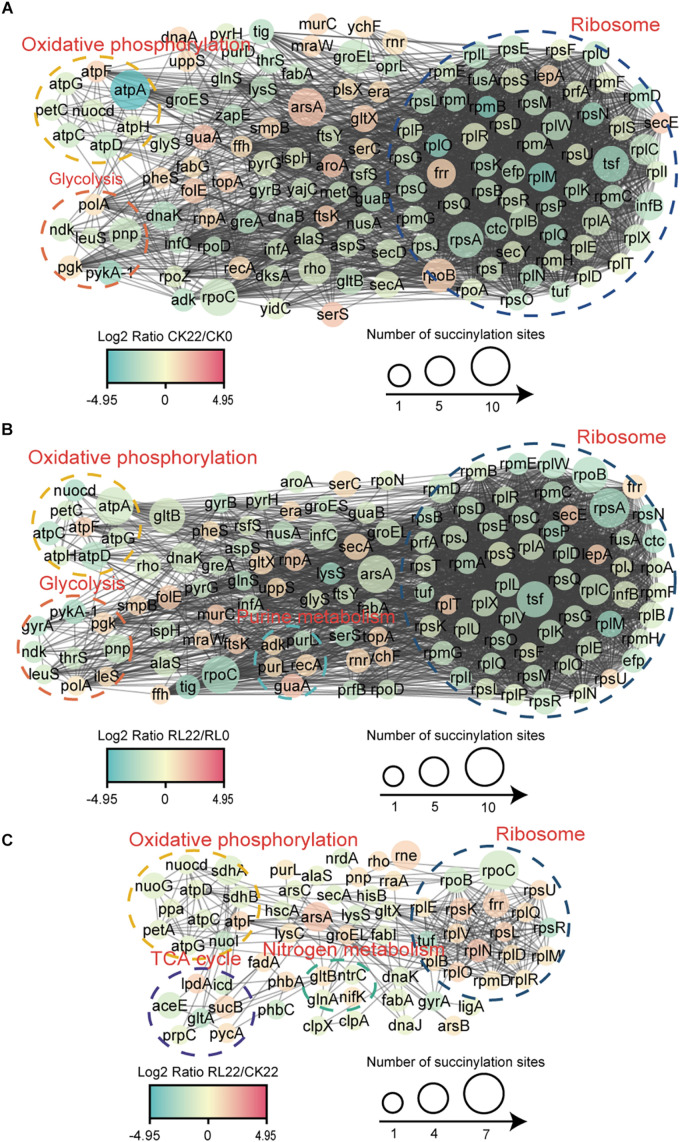
Protein–protein interaction (PPI) network of the succinylated proteins identified in *A. chroococcum*. **(A)** The PPI network of CK22/CK0. **(B)** The PPI network of RL22/RL0. **(C)** The PPI network of RL22/CK22.

To reveal the relationships of succinylated proteins and the molecular mechanisms of the enhancement of nitrogen fixation activity in *A. chroococcum* with RL treatment, the PPI networks were constructed (Figure 7 and Supplementary Table 9).

A total of 135 succinylated proteins interacting with each other in CK22/CK0 were profiled, and the predominant interrelated clusters were oxidative phosphorylation, glycolysis, and ribosomes (Figure 7A), while five succinylated proteins, which were enriched in purine metabolisms, were also present in RL22/RL0 (Figure 7B). In RL22/CK22, succinylated proteins were significantly clustered in TCA cycle and nitrogen metabolism, except in the oxidative phosphorylation and ribosomal complexes (Figure 7C).

**FIGURE 8:**
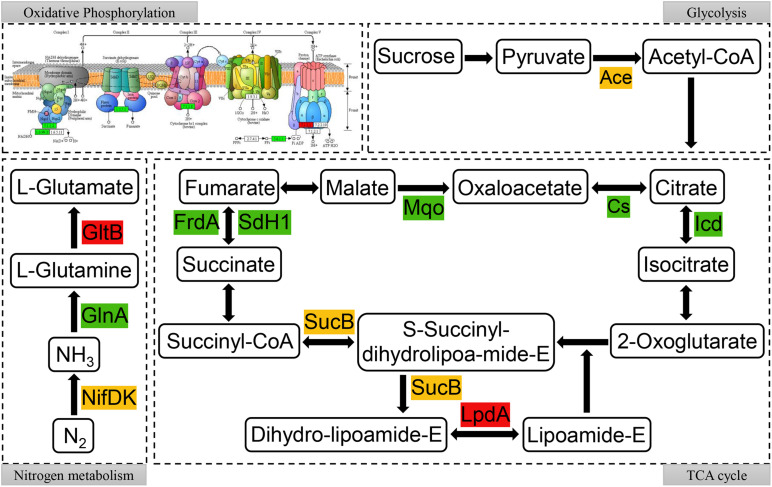
Succinylated proteins in oxidative phosphorylation, glycolysis, nitrogen metabolism, and tricarboxylic acid (TCA) cycle metabolic pathways in RL22/CK22. Different colors represent the upregulated (red), downregulated (green), or up/downregulated (yellow) succinylated proteins.

To further reveal the relationship between succinylated proteins and nitrogen fixation activity, the network involved in the nitrogen fixation process in *A. chroococcum* with RL treatment was mapped, which was based on the enrichment of succinylated proteins in KEGG pathway (Supplementary Figure 2) and the interactions between succinylated proteins. As shown in Figure 8, succinylated proteins were involved in almost all parts of oxidative phosphorylation and TCA cycle. It is noteworthy that there were multiple modification sites of K^Suc^ on the MoFe protein (NifDK) (succinylated at K330, K341, and K433 on NifD and K300 on NifK) and flavodoxin (NifF) (succinylated at K3 and K160) (Supplementary Table 10). Therefore, the level of K^*Suc*^ on NifDK may have a great correlation with the nitrogen fixation activity of *A. chroococcum*.

## Discussion

*Azotobacter* species were model organisms to study nitrogenase and the mechanisms of nitrogen fixation (Baars et al., 2016; Danyal et al., 2016; Rebelein et al., 2018). Meanwhile, *Azotobacter* species is important as free-living, nitrogen-fixing bacteria and potential bacterial biofertilizer (Sumbul et al., 2020; Aasfar et al., 2021). In addition, *A. chroococcum* has other characteristics, such as phosphorus hydrolysis, synthetic indoles, and alginate (Romero-Perdomo et al., 2017; Gawin et al., 2020). RL was mainly produced by *Pseudomonas aeruginosa* (Abbasi et al., 2012) and belonged to biosurfactant, which is capable of inhibiting plant pathogen and improving composting efficiency (Sachdev and Cameotra, 2013; Inès and Dhouha, 2016; Thakur et al., 2021). In this study, we found that RL could significantly enhance the nitrogen-fixing activity of *A. chroococcum*, in which the nitrogen fixation activity was increased by 39.7% with RL (250 mg/l) treatment. Therefore, RL has the potential to improve the nitrogen fixation activity and agricultural production of diazotrophs.

The 4D label-free quantitative proteomic approach is highly sensitive and capable of accurate quantification (Meier et al., 2018; Lesur et al., 2021; Loginov et al., 2021). This approach can detect more low-abundance proteins, but many low-abundance proteins were not identified with western blotting (Kurien and Scofield, 2015). Western blotting qualitatively or semiquantitatively identifies proteins, that is, the corresponding size of protein band has many target proteins (Hnasko and Hnasko, 2015). The size of the differentially succinylated proteins may be higher or lower, which meant that they may counteract each other. Although in this paper the results of western blotting exhibited similar succinylation levels between CK22 and RL22, the detailed proteomic analysis showed differences between CK22 and RL22. K^Suc^ is an important protein PTM that is closely associated with multiple biological processes. LC-MS/MS was used for quantitative proteomics to perform a global succinylation analysis of *A. chroococcum*, and 5,008 succinylation sites in 1,376 proteins were identified. Several reports had shown that PTMs could be changed in response to environmental factors. For example, acetylation of *Escherichia coli* was the most significant process in PTMs cultured in ethanol-enriched media (Soufi et al., 2015), and the level of K^Suc^ in *Bacillus subtilis* was varied in response to different carbon sources (Kosono et al., 2015). We did not find studies of other modifications in diazotrophs, but found quantitative analysis of the modified proteins in other organisms with a fold change of 1.3 (Jiang et al., 2018; Zhang et al., 2019; Li et al., 2020). Therefore, the 1.5-fold change in succinylation is relevant enough in our study. We found that RL could change the enrichment of K^Suc^ in *A. chromococcus*, which indicated that environmental factors played a key role in PTMs. Succinylated proteins were involved in various biological process in *A. chroococcum* and significantly enriched in metabolism. However, most succinylated proteins were associated with the function and activity of enzyme. K^Suc^ played an important role in regulating the activities of enzymes (Ren et al., 2018; Zhou et al., 2019), and we judged that K^Suc^ might be involved in the nitrogen fixation process in *A. chroococcum* by regulating the structure and function of proteins in this study.

Under the optimal conditions, the overall reaction catalyzed by nitrogenase was described as follows: N_2_ + 8H^+^ + 16MgATP + 8e^−^→2NH_3_ + H_2_ + 16MgADP + 16Pi (Ribbe et al., 2014). Therefore, BNF requires large amounts of ATP and e^–^. As nitrogen fixation proceeds, the expression of *glnA* (encoding glutamine synthetase I) and genes in respiration was increased while the TCA cycle was decreased (Sarkar and Reinhold-Hurek, 2014; Shi et al., 2016). In RL22/CK22, the oxidative phosphorylation and TCA cycles contained succinylated proteins, which could provide the ATP and e^–^ in nitrogen fixation process. In addition, there was K^Suc^ enrichment of glutamine synthetase (GlnA) and glutamate synthase (GltB) in glutamate metabolism. The fixed nitrogen by *A. chroococcum* could enter into other metabolic pathways after its assimilation by GlnA and GltB, so we speculate that K^Suc^ was involved in the nitrogen fixation process, which was altered by RL.

The *nifA* is the positively regulated gene for *nif* operon expression in *A. vinelandii* (Mitra et al., 2005). The nitrogenase is composed of Fe protein encoded by *nifH* and MoFe protein encoded by *nifDK* (Einsle et al., 2002). In this study, RL was able to significantly increase the transcript levels of *nifA* and *nifHDK*, which could promote the synthesis of nitrogenase and then enhance the efficiency of nitrogen fixation. Nitrogenase type 1 oxidoreductase is the domain of nitrogenase, and the 4Fe-4S cluster is essential for nitrogenase cofactor assembly (Hu and Ribbe, 2013; Rettberg et al., 2020). Protein domains were significantly enriched in nitrogenase component type 1 oxidoreductase and 4Fe-4S dicluster domain in RL22/CK22. Proteins with these enriched domains play a key role in the synthesis of nitrogenase (Ribbe et al., 2014; Sickerman et al., 2017), so we speculate that K^Suc^ regulates the nitrogen fixation activity of *A. chroococcum* by affecting the activity of nitrogenase. NifDK is the catalytic center of nitrogenase (Spatzal et al., 2011), and the reduced flavodoxin (NifF) is the direct electron donor to Fe protein (Neuer and Bothe, 1985), all of them are the key enzymes that directly affect the nitrogen fixation. Multiple succinylation sites were identified on NifDK and NifF, which indicated that K^Suc^ may play a direct role in the regulation of nitrogen fixation in *A. chroococcum*.

**FIGURE 9:**
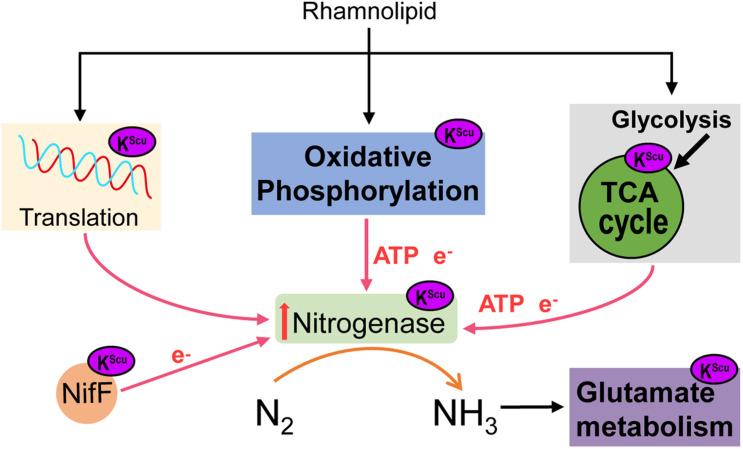
A model of the role of RL in mediating K^Suc^ involved in the regulation process of nitrogen fixation in *A. chroococcum*.

Based on previous studies, we hypothesized a model of K^Suc^ involved in the regulation process of nitrogen fixation in *A. chroococcum* (Figure 9). When *A. chroococcum* cells are treated with RL, RL penetrate the cytoplasm and lead to altered K^Suc^ level. The K^Suc^ could increase the amount of transcript levels of *nifA* gene by modifying nucleic acid transcriptional regulatory proteins, which in turn upregulated the *nifHDK* genes and promoted the nitrogenase synthesis in *A. chroococcum*. On the other hand, succinylated proteins participated in the nitrogen fixation process through oxidative phosphorylation and TCA cycle, which could provide ATP and e^–^ and ensure the smooth progress of nitrogen fixation. At the same time, K^Suc^ modifies the NifDK of nitrogenase and NifF, which may affect the activity of these two enzymes and alter the catalytic process. Finally, nitrogenase converts nitrogen into ammonia, which then enters into other metabolic pathways.

In summary, we hypothesized that K^Suc^ may play a key role in regulating the process of nitrogen fixation. K^Suc^ has a greater impact on protein function than lysine methylation or acetylation (Yang and Gibson, 2019). We have shown that there is NifDK of nitrogenase by K^Suc^. As for the modification sites of K^Suc^ on NifDK influencing nitrogenase activity needs further verification. Therefore, we will analyze the succinylation sites on NifDK to investigate the potential role of K^Suc^ in nitrogen fixation.

## Conclusion

In this study, we found that RL has a positive impact on the nitrogen-fixing activity in *A. chroococcum*. Further studies indicate that RL altered the K^Suc^ level and succinylated proteins were involved in important biological pathways. The study shows that succinylation sites on NifDK may influence nitrogenase activity. To our knowledge, this is the first reported data on K^Suc^ in *A. chroococcum*. Our study provides a foundation for further research on the role of K^Suc^ in regulating biological nitrogen fixation. However, more studies are needed to reveal the effects of succinylated proteins in *A. chroococcum* and to better understand the underlying mechanisms of nitrogen fixation in control of protein succinylation’s ability.

## Data Availability Statement

The raw data supporting the conclusions of this article will be made available by the authors, without undue reservation.

## Author Contributions

XL and YT initiated and designed the research. JL and HP performed the experiments and drafted the manuscript. JL, HY, CW, HL, HZ, PL, and CL analyzed the data. JL, HP, and HY prepared materials. All of the authors read and approved the final manuscript.

## Conflict of Interest

The authors declare that the research was conducted in the absence of any commercial or financial relationships that could be construed as a potential conflict of interest.

## Publisher’s Note

All claims expressed in this article are solely those of the authors and do not necessarily represent those of their affiliated organizations, or those of the publisher, the editors and the reviewers. Any product that may be evaluated in this article, or claim that may be made by its manufacturer, is not guaranteed or endorsed by the publisher.

## Footnotes

1
http://www.ebi.ac.uk/GOA/
