# Supplementary material for: Rhamnolipid Enhances the Nitrogen Fixation Activity of Azotobacter chroococcum by Influencing Lysine Succinylation
Source: Front Microbiol. 2021 Jul 30;12:697963. doi: 10.3389/fmicb.2021.697963 (PMC8360865; doi:10.3389/fmicb.2021.697963)
Supplement: Supplementary file 1 [file Data_Sheet_1.docx]

Supplementary Material

# Supplementary Figures and Tables

## Supplementary Figures


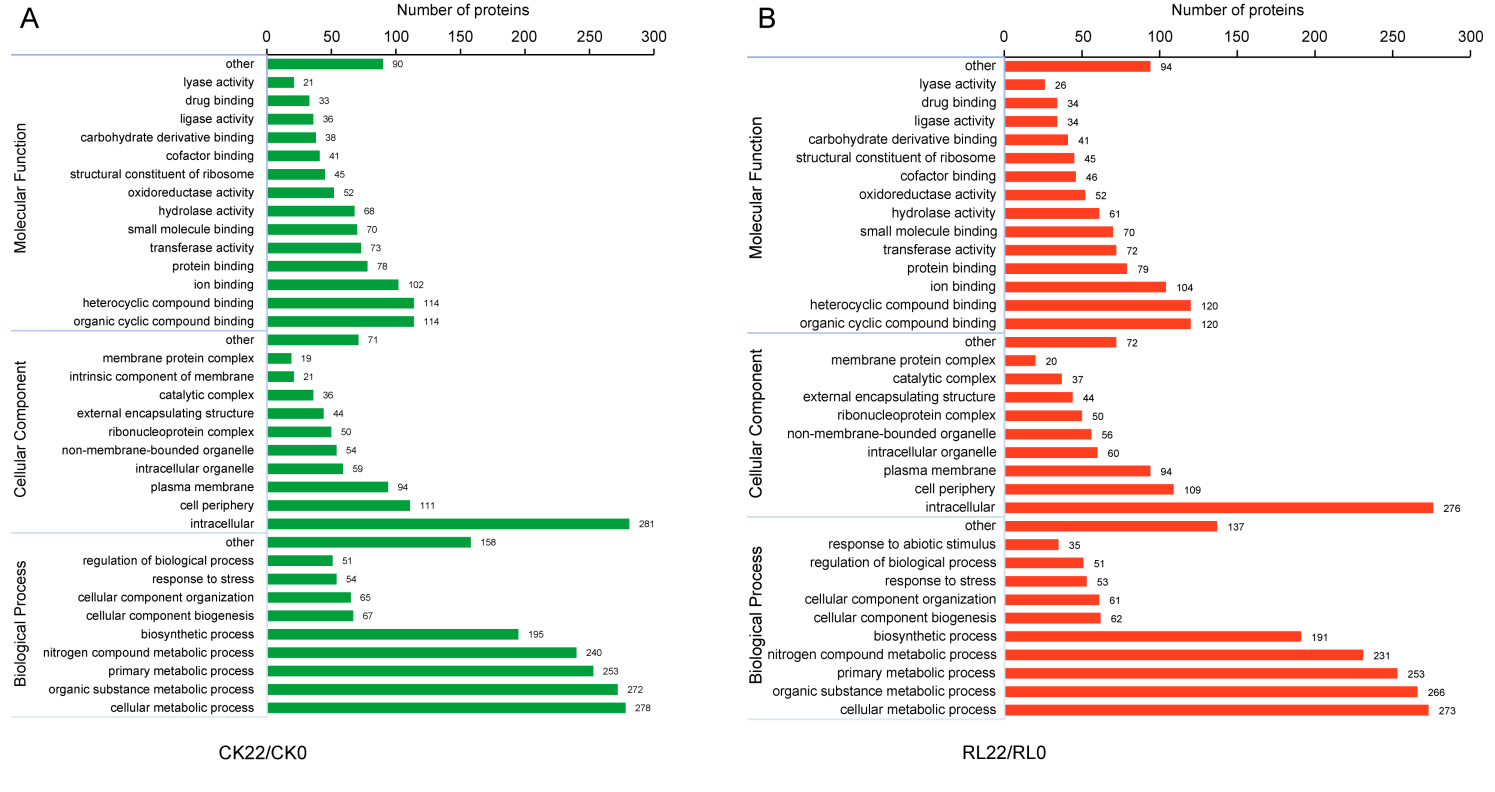


**Supplementary Figure1.** Go functional annotation of succinylated proteins.(A)CK22/CK0. (B) RL22/RL0.


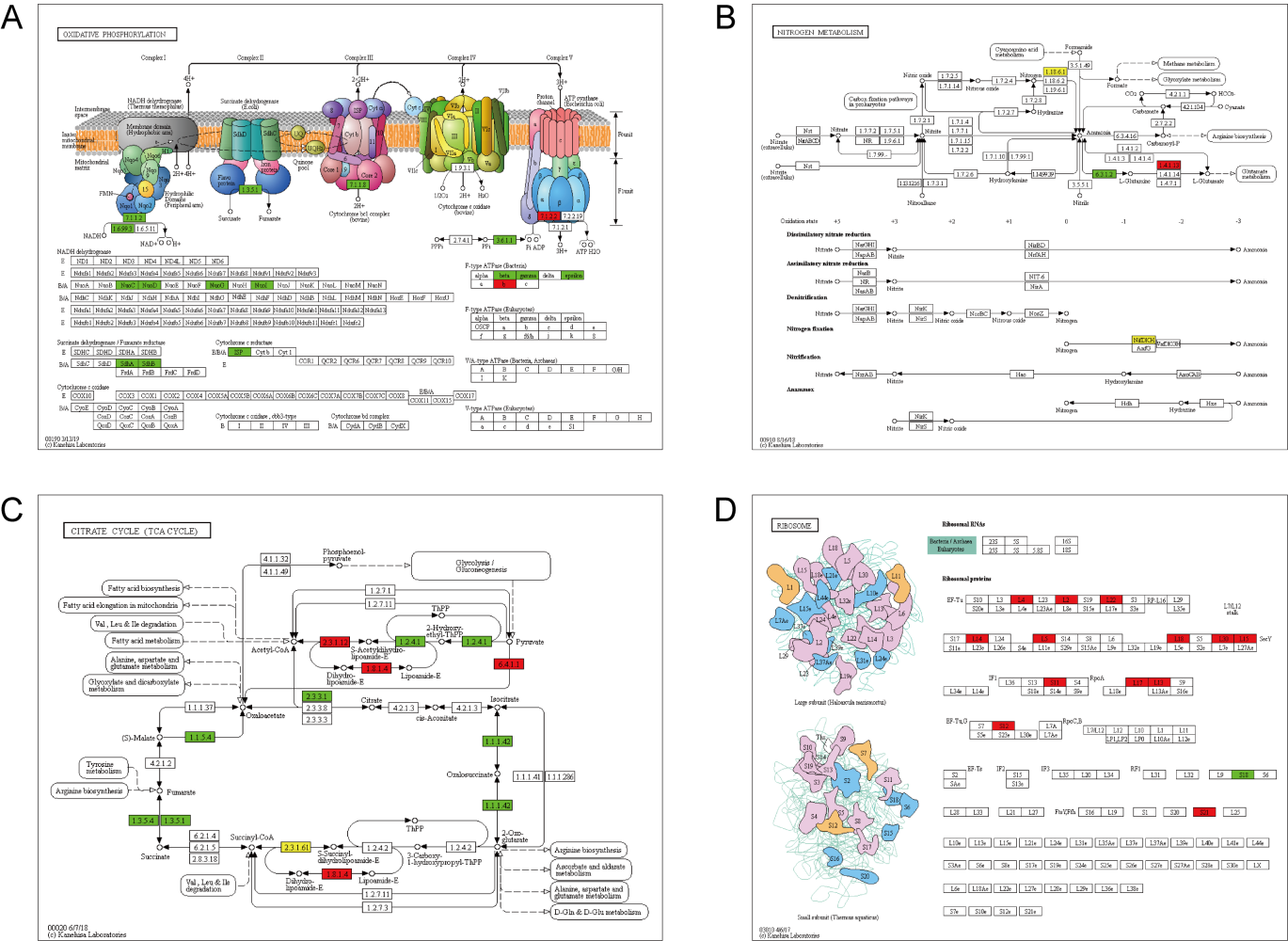


**Supplementary Figure2.** Significant enrichment of succinylated proteins in KEGG pathways in RL22/CK22. (A) Oxidative phosphorylation pathway.(B) Nitrogen metabolism.(C) TCA cycle. (D) Ribosome.

## SupplementaryTables

**Supplementary Table 1.** Real-time PCR primers for *nif* genes.

| Gene | Primer sequences | |
| --- | --- | --- |
|  | Forward primer(5′–3′) | Reverse primer(5′–3′) |
| 16S rRNA | CACTGGAACTGAGACACG | CCGGTGCTTATTCTGTCG |
| *nifA* | ATCCACACCGACTCGGAAG | GCGATGAACGGCATTTCC |
| *nifH* | CATGGAAGCCGAAGACGAG | GAGATGGGGAACTCGGTG |
| *nifD* | TCCAACAAGAAGTCCCAGC | GTGGTGCCGATGTAGTAG |
| *nifK* | GCACCACCCAGGAAGAG | ATCGGGATGTTCAGCTTC |

**Supplemental Table 2.**List of identified lysine succinylated proteins and sites.

**Supplemental Table 3.**Differentially expressed statistics.

**Supplemental Table 4.**The numbers of identified modification sites.

**Supplemental Table 5.** GO and Subcellular Classify of succinylated proteins.

**Supplemental Table 6.**GO Classify of succinylated proteins in CK22/CK0.

**Supplemental Table 7.**GO Classify of succinylated proteins in RL22/RL0.

**Supplemental Table 8.**Enrichment analysis of succinylated proteins.

**Supplemental Table 9.** Protein-protein interaction network for succinylated proteins.

**Supplementary Table10.** The identified succinylated proteins related to the nitrogen fixation system.

| Modified protein | Position | Ratio | Modified sequences |
| --- | --- | --- | --- |
| NifD | 330 | 0.59 | KCEEVIAK(1)YKPEWEAVVAK |
|  | 341 | 0.65 | PEWEAVVAK(1)YRPR |
|  | 433 | 1.55 | FIFQK(1)MGIPFR |
| NifK | 300 | 1.75 | DAPNALSTVLLQPWQLEK(0.969)TKK |
| NifF | 3 | 1.54 | AK(1)IGLFFGSNTGK |
|  | 160 | 2.10 | FVGLALDLDNQSGK(1)TDER |
